# Effect of breathing exercises to prevent pulmonary complications in patients undergoing coronary artery bypass graft surgery: a prospective randomized controlled trials study protocol

**DOI:** 10.3389/fmed.2024.1424291

**Published:** 2025-01-15

**Authors:** Chao Li, Ping Zhang, Zichang Zhang, Delin Qi, Hongli Li

**Affiliations:** Xuanwu Hospital, Capital Medical University, Beijing, China

**Keywords:** preoperative breathing exercises, coronary artery bypass surgery (CABG), pulmonary complications, postoperative recovery, oxygen saturation, six-minute walk test

## Abstract

**Purpose:**

To study the effects of breathing exercises on preventing pulmonary complications in patients undergoing coronary artery bypass graft surgery.

**Methods:**

Observing whether preoperative breathing exercises can reduce the incidence of postoperative pulmonary complications in patients undergoing coronary artery bypass graft surgery; observing whether these exercises can improve postoperative arterial oxygen pressure, oxygen saturation, and the distance walked in a six-minute walk test after surgery; as well as reduce hospital stay duration, lower treatment costs, and improve the quality of life as measured by the Short Form-36 Health Survey (SF-36).

**Design:**

The study population includes patients undergoing coronary artery bypass graft surgery under general anesthesia; the research center is Capital Medical University Xuanwu Hospital; the sample size is 120. Preoperative standardized breathing exercises are utilized, and the incidence of postoperative pulmonary complications, postoperative arterial blood gases, oxygen saturation, six-minute walk test distances, and comparisons of hospital stay durations and costs will be observed.

## Background

Postoperative pulmonary complications are among the most common causes of morbidity and mortality in patients undergoing open-heart surgery and cardiopulmonary bypass. The complications include atelectasis, pulmonary infections such as pneumonia and bronchitis, pleural effusion, pulmonary edema, respiratory insufficiency, exacerbation of chronic lung disease, bronchospasm, and other types of respiratory insufficiency (1, 2). Pulmonary complications lead to an increased length of stay in the Cardiac Intensive Care Unit (CCU), duration of ventilator use, and mortality (3, 4). Coronary artery bypass graft surgery is significant factor in the progression of postoperative pulmonary dysfunction. These effects may persist and result in reductions in lung volume of 25–30% (3.5 months after coronary artery bypass graft surgery (CABG)) (5).

Breathing exercises, as a form of preoperative conditioning, may be an useful approach to reducing the occurrence of postoperative pulmonary complications. With this approach, the patient receives preoperative exercises, which includes balloon-blowing exercises, coughing, and deep breathing, to enhance muscle strength and endurance. Breathing exercises has been widely used to prevent postoperative pulmonary complications in patients undergoing major surgery. It may help improve respiratory muscle activity, re-expand collapsed lungs area, and maintain high pulmonary volumes. However, the evidence supporting its effectiveness is inconclusive (6). In theory, breathing exercises would increase the baseline strength and endurance to a higher level before surgery, resulting in a reduced decrease in respiratory efficiency and therefore a lower risk of developing post-operative pulmonary complications (7–10).

Hence, this study aims to determine whether perioperative breathing exercises can reduce the incidence of postoperative pulmonary complications in patients undergoing CABG surgery.

## Study introduction

### Study purpose

To investigate the effects of breathing exercises on preventing pulmonary complications in patients undergoing coronary artery bypass graft surgery.

### Study content

This randomized controlled trial aims to rigorously evaluate the impact of perioperative breathing exercises on preventing postoperative pulmonary complications (PPCs) and enhancing recovery and patient satisfaction in individuals undergoing coronary artery bypass graft (CABG) surgery. The objectives are designed to provide a detailed and quantifiable assessment of the intervention’s efficacy, safety, and overall impact on.

### Study subjects (including inclusion criteria, exclusion criteria, discontinuation criteria, and termination criteria)

#### Inclusion criteria

1. Patients undergoing elective coronary artery bypass graft surgery under general anesthesia with tracheal intubation.
2. ASA (American Society of Anesthesiologists) classification III.
3. Expected survival time greater than 3 months.
4. Able to cooperate with the observation of adverse events and effectiveness.
5. Patient or their legal representative has signed a written informed consent form.

#### Exclusion criteria

**Figure 1:**
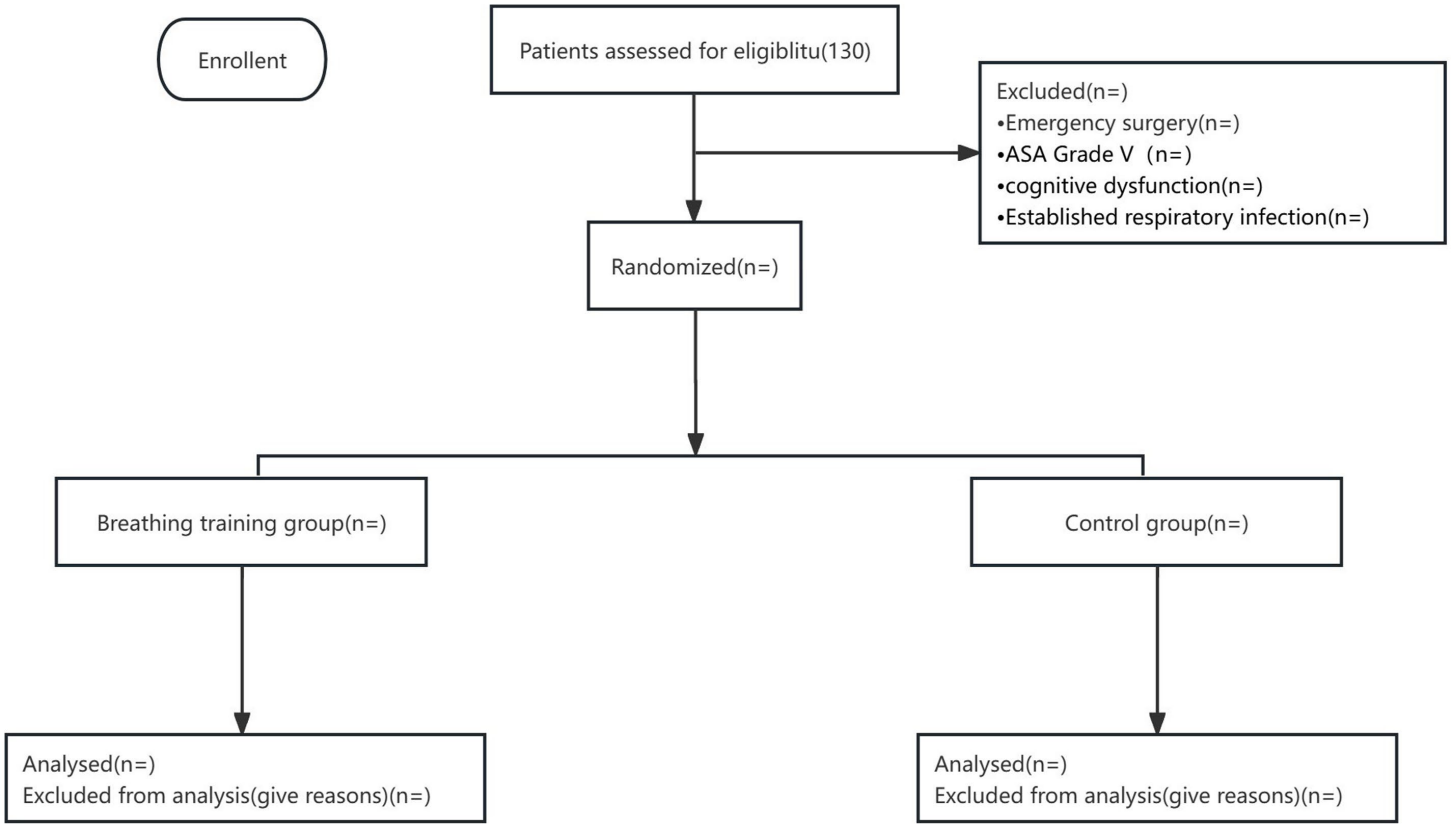
Flow diagram.

1. Patients undergoing emergency surgery.
2. Cognitive impairment.
3. Neuromuscular diseases.
4. History of spontaneous pneumothorax.
5. Coagulation dysfunction.
6. Acute respiratory failure.
7. Confirmed respiratory infection.
8. Uncontrolled general infection.
9. Participation in a clinical drug trial within the past 3 months; patients who refuse to participate in the study (see Figure 1).

#### Discontinuation criteria

The trial will be discontinued if symptoms such as chest tightness or gasping indicative of myocardial infarction or angina occur during the breathing exercises, based on a combined decision by the participant or the research team.

#### Termination criteria

The study will be terminated if serious complications occur during the clinical trial, such as significant bleeding, pulmonary embolism, or severe pulmonary infections. The ethical committee has the authority to terminate or suspend any approved trial if the researcher does not comply with the protocol or applicable regulations, and if the situation is serious or persistent, the researcher’s participation in the clinical trial will be terminated and reported to the regulatory authorities.

## Methods

### Explanation for the choice of comparators

The control group will receive budesonide and ipratropium bromide aerosol inhalation twice a day before operation. This will ensure an optimal comparison with the treatment group.

### Intervention description

**Figure 2:**
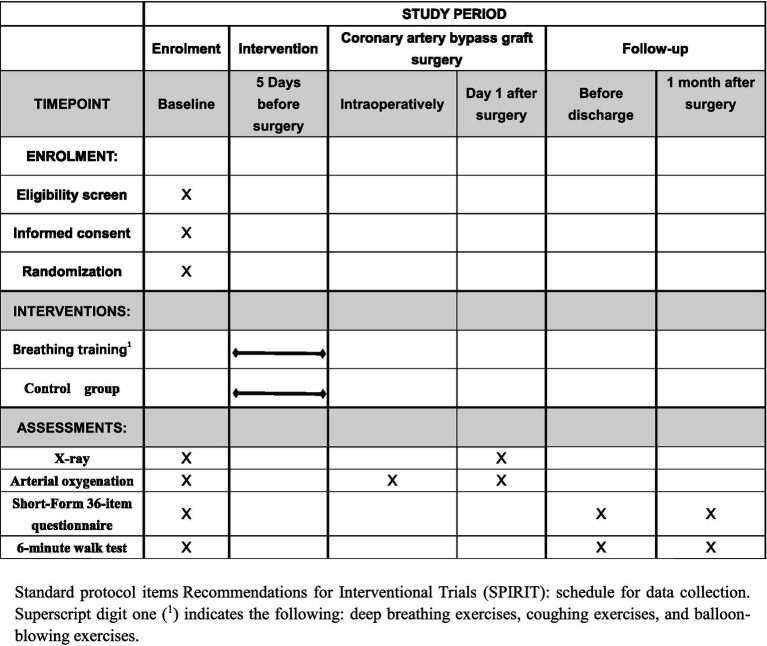
The schedule of enrollment, interventions, and assessments.

The intervention is grounded in the principle that enhanced preoperative respiratory function can reduce the risk of PPCs. Patients in the intervention group in addition to receive budesonide and ipratropium bromide aerosol inhalation twice a day, preoperative breathing exercises also required. Breathing exercises included deep breathing exercises, coughing exercises, and balloon-blowing exercises, for at least 5 days before surgery. Patients will perform 3 to 5 sessions daily, with each session lasting approximately 15 min, at the beginning of each session, healthcare providers will guide patients to ensure they use correct techniques and will monitor tolerance and effectiveness throughout the training. Breathing exercises are designed to strengthen the respiratory muscles and improve lung expansion, thereby potentially reducing the incidence of atelectasis and other complications. When patients in the intervention group were admitted to the hospital, they were provided with brochures and a video that explained the breathing exercises method. The intervention group was conducted as follows (see Figures 1, 2).

- Balloon-blowing exercise

The patient sits down in the chair. Inhale fully through the nose and hold for a full 3-s inhalation. Then exhale through the mouth into the balloon fully and hold the exhalation for 1 s. Cover the balloon immediately with fingers and count it as 1 breath cycle. Finally, replace the balloon immediately. Do this for three consecutive rounds, counted as one set. In each exercises session, complete a total of three sets. Rest for 1 min between sets, which will take approximately 15 min. Repeat this routine three to five times per day.

- Deep breathing and coughing exercises

Deep breathing exercises involve taking deep and long breaths through the nose. The patients inhaled air into their lungs and then held their breath for 3 s before exhaling through the mouth. Coughing exercises were performed to mobilize the lung secretions. Patients rested for 30 s after each cough. Patients in the sitting position perform deep breathing and coughing exercises. The deep breathing and coughing exercises should be repeated 5–10 times daily.

### 6-Minute walk test (6MWT) protocol

- *Preparation*: Ensure that the patient has rested for at least 10 min prior to the test, we use a pulse oximeter to recorded Baseline of SpO₂ and HR, after that the patient will be given instructions on the test procedure, including encouragement to walk as far as possible along a designated 30-meter hallway within the 6-min time frame.
- *During the test:* SpO₂ and HR will be continuously monitored with a portable pulse oximeter to ensure safety. Readings will be recorded at the end of each minute and If SpO₂ drops below 85% or the patient experiences severe dyspnea, dizziness, or chest pain, the test will be paused or terminated according to safety guidelines.
- *End of the test:* At the end of the 6 min, the total distance walked (in meters) will be recorded and final SpO₂ and HR readings will be taken and documented.
- *Post-test monitoring:* After the test, patients will be monitored for 5 min, with SpO₂ and HR recorded each minute until they return to baseline levels.

### Pre-habilitation

Upon admission, patients will be provided with educational materials, including brochures and instructional videos, to ensure they correctly understand the exercise methods and to enhance adherence. Medical staff will monitor the patients’ training progress and provide additional guidance as needed. For at least 5 days before surgery, patients will undergo structured breathing exercises (such as deep breathing and balloon-blowing exercises) aimed at strengthening respiratory muscles, increasing lung capacity, and improving secretion clearance, which are crucial for reducing postoperative pulmonary complications. A team consisting of researchers, nurses, and doctors will collaboratively oversee the prehabilitation process, ensuring comprehensive support and making timely adjustments to enhance the effectiveness of the intervention.

### Surgical procedure

The operation performed is coronary artery bypass graft surgery. At the end of the surgery, patients’ arterial oxygen pressure and oxygen saturation are measured. On the first postoperative day, patients’ arterial oxygen pressure and oxygen saturation are monitored again, along with any occurrences of pulmonary complications (as listed in Table 1) (12–14). Before discharge, the distance walked during a 6-minute walk test and the quality of life scores using the Short Form-36 Health Survey (SF-36) are recorded. Thirty days after discharge, the distance walked in the six-minute walk test and the SF-36 scores are recorded again (15–18).

**Table 1:** Definitions of postoperative pulmonary complications.

Complication	Definition
Respiratory infection	Patient has received antibiotics for a suspected respiratory infection and met one or more of the following criteria: new or charged sputum new or changed lung opacities, fever, white blood cell count>12*109/L.
Respiratory failure	Postoperative PaO2 < 8 kPa (60 mmHg) on room air, a PaO2:FiO2 ratio < 40 kpa (300 mmHg) or arterial oxyhemoglobin saturation measured with pulse oximetry <90% and requiring oxygen therapy.
Pleural effusion	Chest radiograph demonstrating blunting of the costophrenic angle, loss of sharp silhouette of the ipsilateral hemidiaphragm in upright position, evidence of displacement of adjacent anatomical structures or (in supine position) a hazy opacity in one hemithorax with preserved vascular shadows.
Atelectasis	Lung opacification with a shift of the mediastinum, hilum or hemidiaphragm toward the affected area, and compensatory over-inflation in the adjacent non-atelectatic lung.
Pneumothorax	Air in the pleural space with no vascular bed surrounding the visceral pleura
Bronchospasm	Newly detected expiratory wheezing treated with bronchodilators.
Aspiration pneumonitis	Acute lung injury after inhalation of regurgitated gastric contents.
Pneumonia	Chest radiograph with at least one of the following: infiltrates, consolidation, cavitation; and at least one of the following: fever (>38°C) with no other recognized cause, white cell count >12 × 109/L or < 4 × 109/L, >70 years old with altered mental status with no other recognized cause; and at least two of the following: purulent sputum or increased respiratory secretions, cough or dyspnea or tachypnoea, rales or bronchial breath sounds, worsening gas exchange.

## Sample size

The sample size calculation is based on the expected incidence of postoperative pulmonary complications (PPC) (5, 22). Using the formula for estimating a proportion with specified absolute precision: n = (Z^2^ * p * (1−p)) / E^2^, where n is the sample size, Z is the z-value corresponding to the desired confidence level (1.96 for 95% confidence), p is the estimated incidence of PPC in the population, and E is the desired margin of error. For this study, assuming a PPC incidence of 15% based on previous studies, and aiming for a margin of error of 5%, the calculation yields a sample size of approximately 108 patients. Considering a potential dropout rate of 10%, the total sample size was adjusted to 120 patients.

### Main and secondary endpoint

#### Main endpoint

Six-Minute Walk Distance (6MWD) at 1 month postoperatively: the primary endpoint is the measurement of 6MWD at 1 month after surgery. This time point has been chosen as it provides an ideal period for assessing improvements in lung function, endurance, and the effects of rehabilitation interventions. Studies have indicated that the 6MWD at 1 month is strongly correlated with patients’ long-term exercise tolerance and pulmonary recovery. Additionally, this point allows for the evaluation of the sustained impact of preoperative respiratory training on patients’ recovery during the home rehabilitation phase.

#### Secondary endpoints

- 6MWD before discharge: This endpoint will provide an immediate measure of the patient’s exercise capacity before leaving the hospital, allowing for comparison with the one-month follow-up to evaluate progress (19–21).
- Arterial Oxygen Pressure (PaO_2_) and Oxygen Saturation (SpO_2_): These indicators will be measured to assess respiratory efficiency and oxygenation status, both critical markers for recovery.
- Incidence of Postoperative Pulmonary Complications: Tracking pulmonary complications, such as atelectasis, pneumonia, and pleural effusion, will allow assessment of respiratory health and the effectiveness of preoperative interventions.
- FEV₁ (Forced Expiratory Volume in one second) and FVC (Forced Vital Capacity): Conduct a baseline pulmonary function test before starting the breathing exercise intervention to obtain initial FEV₁ and FVC values. We will record each patient’s FEV₁ and FVC values, calculating changes between the baseline and post-intervention tests. These data will serve as secondary outcome measures to further evaluate the potential improvements in lung function from the breathing exercises (9, 10).
- Length of Hospital Stay: This parameter will be recorded as an indicator of the recovery speed, which is also influenced by respiratory health and complications.
- Total Treatment Cost: By analyzing the total cost, the study will evaluate the economic impact of the intervention and its cost-effectiveness in reducing complications and supporting recovery.

## Data management

Statistical analysis is performed using SPSS version 25.0. Quantitative data following a normal distribution are expressed as mean ± standard deviation, while non-normally distributed quantitative data are expressed using medians (interquartile range). Categorical data are expressed as frequencies or percentages. The Kolmogorov–Smirnov test is used to analyze continuous data to assess the normality of their distribution. Baseline characteristics between groups are compared using independent *t*-tests, Mann–Whitney U tests, chi-square tests, or Fisher’s exact tests. The main endpoints are analyzed using chi-square tests to compare differences between the two groups. Efficacy rates, remission rates, anxiety, severe pain, psychiatric outcomes (mania, psychiatric and dissociative symptoms), and incidence of complications are analyzed using chi-square or Fisher’s exact tests. Postoperative MADRS scores are compared between groups using one-way analysis of variance (ANOVA), with the Hodges-Lehmann method used to estimate the 95% confidence intervals (CIs) for differences between groups. Logistic regression analysis is employed to estimate the interaction effects in subgroup analyses. Sensitivity analysis is used to determine the statistical nature of missing data. Missing data from patients who did not complete any assessment post-operatively are imputed based on baseline data. However, data missing due to early discharge are estimated based on the last observed assessment after surgery. Both primary and secondary outcomes are reported with risk ratios and their 95% CIs.

A two-sided *p*-value of 0.05 is considered statistically significant for primary outcomes. To prevent type I errors, the Holm-Bonferroni method is used to adjust for multiple comparisons in secondary outcomes.

## Feasibility analysis

1. Xuanwu Hospital’s Department of Cardiac Surgery performs over 200 coronary artery bypass graft surgeries annually, sufficiently meeting the case requirements for clinical trials.
2. Xuanwu Hospital’s Department of Cardiac Surgery is equipped with advanced monitoring devices, fully capable of supporting surgical and clinical research needs.

## Study quality control

1. Strict inclusion criteria.
2. Strict adherence to the study design for sampling and conducting visits.
3. Standardized preoperative management with postoperative follow-ups conducted by the same external physician.
4. Rigorous data collection according to the study protocol.

## Study risks and safety measures

The main risks of this study involve chest tightness and pain potentially induced during the breathing exercises, which may suggest symptoms of angina or myocardial infarction. The trial will be stopped immediately if such symptoms occur, with medical treatment provided, and surgical intervention if necessary. Additional risks include post-surgical complications such as atelectasis, pulmonary infections, and hypoxemia. If any research-related injuries or diseases occur, participants should immediately contact their physician for necessary medical advice and treatment. If pre-surgery insurance is purchased, participants should immediately contact the research physician and the insurance company to provide necessary medical advice and compensation.

## Recruitment of study participants

Participants are recruited during the diagnostic and treatment process by the researcher. Three days before being enrolled in the study, the researcher will inquire and record the patient’s medical history, and perform physical examinations and anesthesia assessments. If the patient meets the inclusion criteria and voluntarily agrees to participate, they will sign an informed consent form.

## Rights, benefits, and compensation for participants

1. Informed consent acquisition: Researchers will explain the benefits of pre-surgical breathing training to the participants, who may voluntarily decide to participate. Researchers will report all relevant events to the participants so they can decide at any time whether to continue participating.
2. Participants and society may benefit from this study. Previous research has shown mixed results regarding whether pre-surgical breathing training reduces the incidence of post-surgical pulmonary complications. This study aims to explore the impact of pre-surgical pulmonary training on post-surgical pulmonary complication rates. Additionally, during the follow-up, patients will be monitored regularly by professionals, receiving free professional consultations.
3. Participants will receive quality medical care during the study. Participants can refuse to participate or withdraw at any time during the study without affecting their relationship with their doctors or losing any medical benefits.
4. If any surgical-related risks occur during the study period, appropriate compensation will be provided through surgical liability insurance if pre-surgery insurance is purchased.

## Participant privacy and confidentiality

All information related to participants, including identity, medical history, health status, physical examinations, and laboratory results, will be kept confidential within the limits allowed by law. Only authorized researchers, ethics committees, and relevant regulatory bodies can access participants’ records related to this study to verify the authenticity and accuracy of the collected data. However, personal details of the participants will not be involved. Participants’ names will not appear in any public documents or reports related to this study.

## Publication or disclosure of study results

This study complies with the Declaration of Helsinki. Regardless of whether the study results are negative, inconclusive, or positive, they will be published or disclosed.

### Study implementation conditions and personnel

1. The study will be conducted in the operating room with anesthesia and surgery performed in the cardiac ward and cardiac care unit, including pre-surgical assessments, signing of informed consent, and post-operative follow-ups.
2. The surgery is performed through a median sternotomy. For CABG, harvesting the left or right internal mammary artery is a routine procedure, but this may impact the function of the phrenic nerve, leading to restricted diaphragmatic movement and increasing the risk of postoperative respiratory difficulties. Coronary artery bypass surgery is typically performed under general anesthesia, with endotracheal intubation and mechanical ventilation to maintain respiration. During general anesthesia, anesthetic drugs, especially opioids, may suppress the patient’s spontaneous breathing, leading to early postoperative hypoventilation. This suppressive effect may persist for several hours postoperatively, affecting airway clearance. General anesthetics can reduce the patient’s cough reflex and impair the ciliary activity of airway mucosa, which decreases the ability to clear mucus, thereby increasing the risk of atelectasis and pulmonary infections. During anesthesia, as the patient is in the supine position and on mechanical ventilation, functional residual capacity (FRC) may decrease, causing partial alveolar collapse. This reduction in lung volume may persist even postoperatively, affecting ventilation efficiency. During anesthesia, all patients receive an oxygen concentration of 40–50% during inspiration, with a tidal volume of 6–8 mL/kg and a positive end-expiratory pressure (PEEP) of 4–6 cm H₂O. Lung recruitment maneuvers (such as temporarily increasing PEEP and tidal volume) may be employed intraoperatively to reopen collapsed alveoli, particularly when switching to cardiopulmonary bypass (CPB) after prolonged mechanical ventilation. After CPB initiation, ventilation is usually reduced or even paused, as the CPB machine takes over oxygenation and carbon dioxide removal. During prolonged CPB, intermittent low tidal volume ventilation (e.g., 2–4 breaths per minute) may be used to keep some alveoli open and reduce the risk of atelectasis. Before ending CPB, mechanical ventilation is typically resumed, along with lung recruitment maneuvers, gradually increasing tidal volume and PEEP to reopen collapsed alveoli. After CPB, the mechanical ventilation system is reconnected, and tidal volume and PEEP are gradually increased to preoperative levels. The anesthesiologists and surgeons performing the anesthesia and surgery are experienced deputy chief physicians of anesthesiology and chief physicians of cardiac surgery, proficient in cardiac surgery anesthesia, coronary artery bypass graft surgery, and postoperative management, ensuring patients smoothly transition through the perioperative period.
3. Ventilator Weaning Protocol: The goal of ventilator weaning is to ensure safe and gradual transition from mechanical ventilation to independent breathing. Weaning should be individualized based on the patient’s respiratory capacity, underlying conditions, and postoperative progress. The patient should meet the following criteria before initiating weaning: (1) Hemodynamic Stability: No significant hypotension or requirement of high-dose vasopressors. (2) Adequate Oxygenation: Arterial oxygen saturation (SpO₂) > 90% on FiO₂ < 0.4, PaO₂ > 60 mmHg, and a PaO₂/FiO₂ ratio > 150. (3) Stable Respiratory Parameters: Respiratory rate < 35 breaths per minute, tidal volume > 5 mL/kg ideal body weight, and minimal secretions. (4) Mental Status: Awake, responsive, and able to follow commands. A spontaneous breathing trial (SBT) is typically the first step in assessing readiness for extubation: (1) Mode: Switch to a low-level pressure support mode (e.g., PSV or CPAP). (2) Duration: Conduct SBT for 30–120 min, monitoring for signs of respiratory distress. (3) Evaluation: Assess respiratory rate, heart rate, blood pressure, and SpO₂ continuously. (4) Failure Criteria: If patient exhibits tachypnea (>35 breaths per minute), hypoxemia (SpO₂ < 90%), or increased work of breathing, terminate SBT and resume ventilator support. Repeat trial after stabilizing parameters. Once the patient successfully completes SBT: (1) Cough and Airway Protection: The patient should have a strong cough reflex and be able to clear secretions. (2) Minimal Sedation: Patient is alert and can protect their airway. (3) Hemodynamic and Respiratory Stability: No significant respiratory or hemodynamic compromise during SBT. After extubation: (1) Frequent Monitoring: Check respiratory rate, oxygen saturation, and blood gases every 1–2 h. (2) Supportive Measures: Provide supplemental oxygen if necessary and continue respiratory exercises (11).
4. Rehabilitation Procedures: Rehabilitation following extubation is essential for enhancing lung function, preventing atelectasis, and promoting overall recovery. The protocol includes respiratory exercises, mobility, and physical therapy components. Respiratory Rehabilitation are Focuses on lung expansion, secretion clearance, and respiratory muscle strengthening: (1) Deep Breathing Exercises: Encourage the patient to perform deep breathing exercises 5–10 times per hour. This exercise improves lung volume and prevents atelectasis. (2) Coughing Exercises: Assist patients in coughing to mobilize and clear secretions. Splint the incision site with a pillow if necessary to reduce discomfort. (3) Balloon-Blowing Exercise: balloon-blowing helps strengthen respiratory muscles and improve lung volume. (4) Nebulization and Humidification: Provide nebulized saline or bronchodilators to maintain airway moisture and facilitate secretion clearance. Early mobilization is critical to improve circulation, prevent thromboembolism, and promote overall recovery: (1) Bed Mobility and Passive Range of Motion Exercises: Initiate passive range of motion exercises in the ICU, followed by assisted bed mobility and limb movements. (2) Sitting and Standing: Aim to get the patient sitting on the edge of the bed within 24 h of extubation, if stable. Gradually progress to standing with support. (3) Walking: Begin supervised walking as soon as the patient can tolerate. Start with short distances, progressively increasing as tolerated to reach 5–10 min of walking per session, aiming for three to four sessions per day.
5. Researchers have obtained Good Clinical Practice (GCP) training certificates.
6. Researchers have undergone rigorous training and are readily available for consultations regarding safety issues.
